# Mechanisms of glabridin inhibition of integrin α_IIb_β_3_ inside-out signals and NF-κB activation in human platelets

**DOI:** 10.1186/s13020-023-00779-9

**Published:** 2023-06-10

**Authors:** Wei-Chieh Huang, Thanasekaran Jayakumar, Joen-Rong Sheu, Chih-Wei Hsia, Chih-Hsuan Hsia, Ting-Lin Yen, Chao-Chien Chang

**Affiliations:** 1 Graduate Institute of Medical Sciences, College of Medicine, Taipei Medical University, Taipei, 110 Taiwan; 2 Department of Ecology and Environmental Sciences, Pondicherry University, Puducherry, 605014 India; 3 Translational Medicine Center, Shin Kong Wu Ho-Su Memorial Hospital, Taipei, 111 Taiwan; 4 Department of Medical Research, Cathay General Hospital, Taipei, 106 Taiwan; 5 Department of Cardiovascular Center, Cathay General Hospital, Taipei, 106 Taiwan; 6 School of Medicine, College of Medicine, Fu Jen Catholic University, New Taipei City, 242 Taiwan; 7 Department of Pharmacology, School of Medicine, College of Medicine, Taipei Medical University, Taipei, 110 Taiwan

**Keywords:** Glabridin, Human platelets, α_IIb_β_3_ inside-out, NF-κB, Thromboembolic lungs, Platelet plug formation

## Abstract

**Background:**

Platelets play a crucial role in cardiovascular diseases (CVDs) and are activated by endogenous agonists like collagen. These agonists initiate signal transduction through specific platelet receptors, resulting in platelet aggregation. Glabridin, a prenylated isoflavonoid found in licorice root, is known for its significance in metabolic abnormalities. Glabridin has been observed to inhibit collagen-induced platelet aggregation, but the precise mechanisms, specifically concerning NF-κB activation and integrin α_IIb_β_3_ signaling, are not yet fully understood.

**Methods:**

In this study, platelet suspensions were prepared from healthy human blood donors, and the aggregation ability was observed using a lumi-aggregometer. The inhibitory mechanisms of glabridin in human platelets were evaluated through immunoblotting and confocal microscopy. The anti-thrombotic effects of glabridin were assessed by histological analysis of lung sections in acute pulmonary thromboembolism and by examining fluorescein-induced platelet plug formation in mesenteric microvessels in mice.

**Results:**

Glabridin inhibited integrin α_IIb_β_3_ inside-out signals such as Lyn, Fyn, Syk, and integrin β_3_ activation and NF-κB-mediated signal events, with similar potency to classical inhibitors BAY11-7082 and Ro106-9920. Glabridin and BAY11-7082 inhibited IKK, IκBα, and p65 phosphorylation and reversed IκBα degradation, while Ro106-9920 only reduced p65 phosphorylation and reversed IκBα degradation. BAY11-7082 reduced Lyn, Fyn, Syk, integrin β_3_, phospholipase Cγ2 and protein kinase C activation. Glabridin reduced platelet plug formation in mesenteric microvessels and occluded vessels in thromboembolic lungs of mice.

**Conclusion:**

Our study revealed a new pathway for activating integrin α_IIb_β_3_ inside-out signals and NF-κB, which contributes to the antiplatelet aggregation effect of glabridin. Glabridin could be a valuable prophylactic or clinical treatment option for CVDs.

**Supplementary Information:**

The online version contains supplementary material available at 10.1186/s13020-023-00779-9.

## Background

Cardiovascular diseases (CVDs) are the leading cause of mortality worldwide, and arterial thrombosis can cause the development of CVDs such as myocardial infarction, venous thromboembolism, atherosclerosis, and even ischemic stroke [1]. Platelets, anucleate blood cells, are key in the development of arterial thrombosis [2]. When vascular subendothelial proteins (i.e., collagen) are exposed due to injury, platelets move, adhere at the injury site, and then initiate and amplify platelet aggregation, a point that conventionally marks the start of intraluminal thrombosis [3]. Platelet receptors initiate intraplatelet signaling pathways by secreting granules containing adenosine diphosphate, Ca^2+^, and fibrinogen, which activates platelet integrin α_IIb_β_3_ and enables platelet aggregation [4]. In its resting state, integrin α_IIb_β_3_ in platelets is present in a low activation state and is unable to interact with specific ligands such as fibrinogen. However, platelet activation induced by various agonists triggers a conformational change in integrin α_IIb_β_3_, facilitating its binding to ligands and leading to platelet aggregation [1].

The NF-κB signaling pathway plays a critical role in the regulation of various cellular events, such as inflammatory and vascular pathological responses [5]. The predominant form of pleiotropic NF-κB is an inactive cytoplasmic complex consisting of p50 and p65 subunits, which tightly bind to the inhibitors of κB (IκB) proteins. The activation of NF-κB occurs when the IκBα protein is phosphorylated by the IκB kinase (IKK) complex, and the NF-κB complex subsequently translocate from the cytosol into the nucleus to regulate gene expression. Platelets lack a nucleus, but they contain many functional transcription factors, including NF-κB, that participate in platelet activation [6]. However, the functioning of NF-κB signaling in platelets remains partially understood, unlike in nucleated cells.

**Fig. 1:**
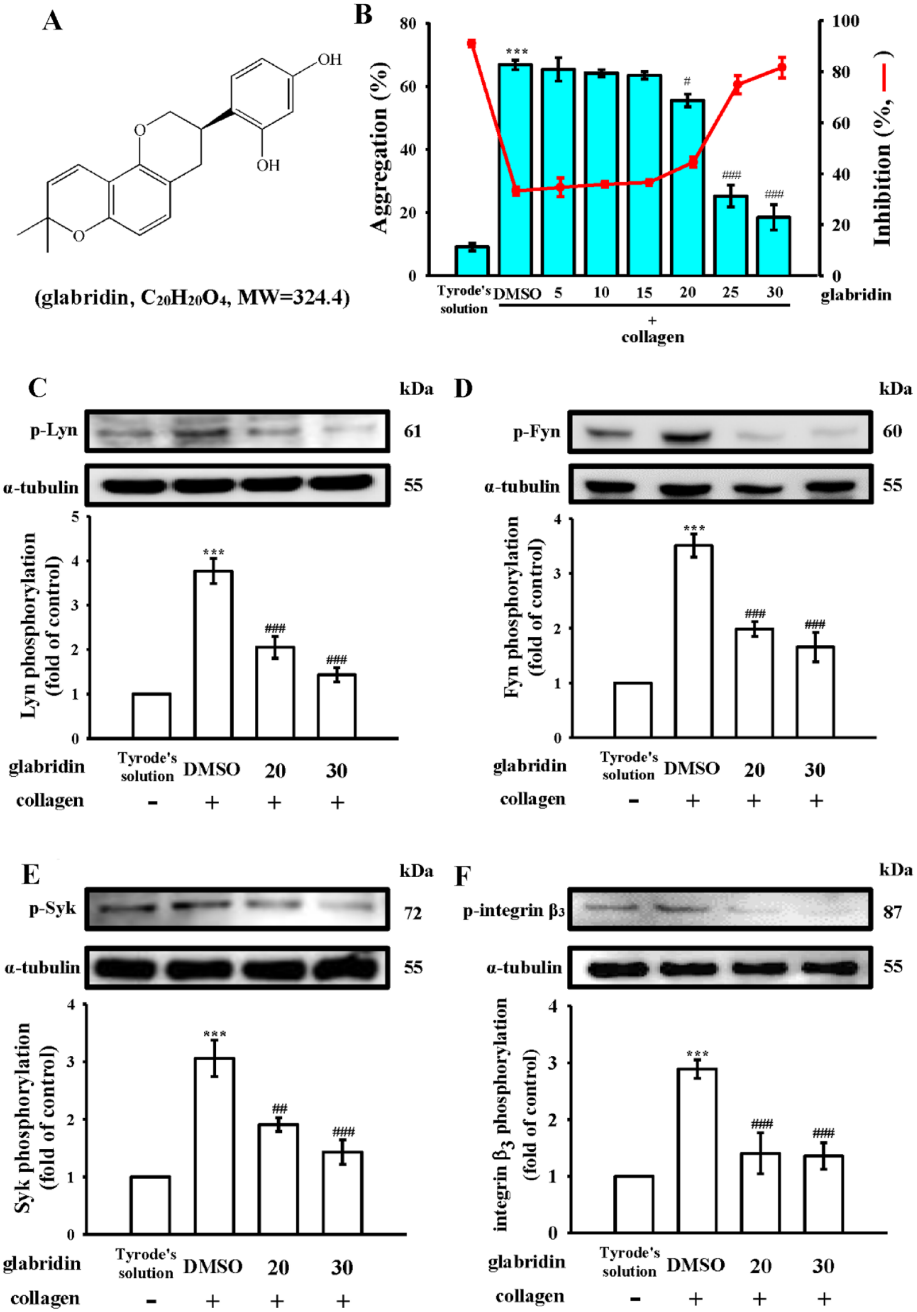
Inhibitory profiles of glabridin for platelet aggregation and Lyn, Fyn, Syk and integrin β_3_ activation stimulated by collagen in human platelets. **A** Chemical structures of glabridin (C_20_H_20_O_4_). **B** Washed human platelets (3.6 × 10^8^ cells/mL) were preincubated with a solvent control (0.1% DMSO) or glabridin (5–30 µM), followed by the addition of collagen (1 µg/mL) to stimulate platelet aggregation. For further experiments, washed platelets were preincubated with a solvent control (0.1% DMSO) or glabridin (20 and 30 µM), followed by the addition of collagen (1 µg/mL) to stimulate **C** Lyn, **D** Fyn, **E** Syk, and **F** integrin β_3_ phosphorylation. Data are presented as mean ± standard error of the mean (*n* = 4). ***P < 0.001, compared with the resting control (Tyrode’s solution); ^##^*P* < 0.01 and ^###^*P* < 0.001, compared with the 0.1% DMSO-treated group

In most traditional Chinese herbal formulas, licorice is used as a guide drug with other herbs in a single prescription to enhance the efficacy of the other herbs, reduce toxicity, and improve flavor [7]. Glabridin (Fig. 1A) is a prenylated isofla vonoid found in licorice root, that is reported to reduce antimetabolic abnormalities (i.e., obesity and diabetes) and possess antiviral, antimicrobial, and estrogen-like properties. In addition, glabridin protects the nervous system and has anticancer and antiosteoporotic properties [8]. Clinical studies have reported that glabridin effectively decreases lipid and glucose levels in patients with metabolic diseases [9, 10]. Dietary supplementation of glycyrrhizin-free licorice root extract containing 60 mg of glabridin in healthy individuals for 6 months reduced plasma low-density lipoprotein oxidation by 20%, indicating that glabridin might be promising in the treatment of atherosclerosis and CVDs [9]. Conversely, licochalcone, a bioactive compound in licorice root, was reported to exhibit antiplatelet activity through inhibition of phospholipase Cγ2 (PLCγ2)/protein kinase C (PKC) and mitogen-activated protein kinase (MAPK) activation in human platelets [11]. Chung et al. [12] found that glabridin most strongly inhibits collagen-stimulated human platelet aggregation and moderately inhibits arachidonic acid (AA) stimulation but has no effect on thrombin or U46619 stimulation, whose mechanisms may be mediated by inhibition of PLCγ2/PKC and phosphoinositide 3-kinase/Akt/glycogen synthase kinase-3β activation. Concurrently, they found that glabridin clearly reduces pulmonary thromboembolism without prolonging bleeding time as compared with aspirin [12]. Therefore, glabridin may have therapeutic potential against thromboembolic-related diseases. Although that study examined the inhibitory effects of glabridin, its mechanisms in platelet activation remain not fully understood, especially regarding NF-κB activation or with other signals such as integrin α_IIb_β. Therefore, we addressed these issues in this study.

## Materials and methods

### Reagents and materials

Glabridin (≥ 98%), BAY11-7082 (3-[(4-methylphenyl)sulfonyl]-(2E)-propenenitrile), and Ro106-9920 (6-(phenylsulfinyl)-tetrazolo[1,5-b]pyridazine) were purchased from Cayman Chemical (Ann Arbor, MI, United States). ADP, collagen (type I), dimethyl sulfoxide (DMSO), bovine serum albumin (BSA), heparin, ethylenediaminetetraacetate (EDTA), phenylmethylsulfonyl fluoride (PMSF), sodium orthovanadate, sodium pyrophosphate, aprotinin, leupeptin, NaF, and paraformaldehyde (PFA) were purchased from Sigma-Aldrich (St. Louis, MO, United States). Phospho-Syk (Tyr^525^/Tyr^526^) polyclonal antibodies (pAbs) were purchased from Affinity Biosciences (Cincinnati, OH, United States). Integrin β_3_ (phospho Tyr^773^) pAb was purchased from GeneTex (Irvine, CA, United States). Anti-phospho-(Ser) PKC substrate, anti-IκBα (44D4), and anti-phospho-NF-κB p65 (Ser^536^) pAbs, anti-phospho-IκBα (Ser^32/36^; 5A5), and phospho-IKKα/β (Ser^176/180^; 16A6) monoclonal antibodies (mAbs) were purchased from Cell Signaling Technology (Beverly, MA, United States). Anti-phospho PLCγ2 and anti-Lyn (phospho Y^507^) mAbs, and anti-Fyn (phospho Y^530^) pAb were obtained from Abcam (Cambridge, United Kingdom). Protein assay dye reagent concentrate was purchased from Bio-Rad Laboratories (Hercules, CA, United States). CF488A and CF405M dyes were obtained from Biotium (Hayward, CA, United States). Anti-α-tubulin mAb was purchased from Santa Cruz Biotechnology (Santa Cruz, CA, United States). Hybond-P polyvinylidene difluoride membranes, enhanced chemiluminescence Western blotting detection reagent, horseradish peroxidase (HRP)-conjugated donkey anti-rabbit immunoglobulin G (IgG), and sheep anti-mouse IgG were obtained from Amersham (Buckinghamshire, United Kingdom). Glabridin was dissolved in 0.1% DMSO and stored at 4 °C for experiments.

### Human platelet preparation and aggregation study

This study was conducted in accordance with the ethical principles of the Declaration of Helsinki and was approved by the Institutional Review Board of Taipei Medical University (TMU-JIRB-N201812024). All human participants in this study provided informed consent. Healthy human blood donors were used to prepare platelet suspensions that were mixed with an acid–citrate–dextrose solution (9:1, v/v), following the method described in another study [13]. After centrifugation, the platelet-rich plasma (PRP) supplemented with EDTA (2 mM) and heparin (6.4 U/mL) was incubated for 5 min and then centrifuged again. The platelet pellets were suspended and centrifuged, and finally suspended in Tyrode’s solution containing BSA (3.5 mg/mL) and Ca^2+^ (1 mM). The platelets were counted using a Coulter counter (Beckman Coulter, Miami, FL, United States). Washed platelets (3.6 × 10^8^ cells/mL) were preincubated with a solvent control (0.1% DMSO) or glabridin (5–30 µM) for 3 min before being stimulated with collagen (1 µg/mL). Aggregation ability was observed using a lumi-aggregometer (Payton, Scarborough, Ontario, Canada) [14]. The degree of platelet aggregation was defined as the percentage of the platelet aggregation observed in the control group (the group treated with Tyrode’s solution) in light transmission units.

### Immunoblotting

Washed platelets (1.2 × 10^9^ cells/mL) were preincubated with a solvent control (0.1% DMSO), glabridin (20 and 30 µM), or other reagents for 3 min, followed by stimulation with collagen (1 µg/mL) for 6 min. The platelet suspensions were lysed using 200 µL of lysis buffer (aprotinin 10 µg/mL, PMSF 1 mM, leupeptin 2 µg/mL, NaF 10 mM, sodium orthovanadate 1 mM, and sodium pyrophosphate 5 mM) for 1 h. The targeted proteins were electrophoretically separated using a 12% SDS-PAGE and the separated proteins were transferred using semidry transfer (Bio-Rad, Hercules, CA, United States) and then blocked using TBST (10 mM Tris-base, 0.01% Tween 20, and 100 mM NaCl) containing 5% BSA for 1 h. A Bradford protein assay (Bio-Rad, Hercules, CA, United States) was performed to quantify protein concentrations. The membranes were incubated with their respective primary antibodies (diluted 1:1000 in TBST) and then with HRP-conjugated anti-mouse or anti-rabbit IgG (diluted 1:5000 in TBST) for 1 h. The intensity of protein bands was calculated using a video densitometer and BioProfil BioLight software, v2000.01 (Vilber, Marne-la-Vallée, France). Relative protein expression was calculated after normalization to the expression of the total protein of interest.

### Confocal laser fluorescence microscopy

Per the method described by Crosby and Poole [15], platelets were immunostained to examine using confocal microscopy assay. Briefly, resting or collagen-activated platelets were fixed in 4% (v/v) paraformaldehyde on poly-l-lysine-coated coverslips for 1 h. Platelets were then permeabilized in 0.1% triton X-100 and incubated with 5% BSA in phosphate-buffered saline (PBS) for 1 h before staining. To observe targeted proteins, platelets were stained with their respective primary antibodies for 24 h. After washing with PBS, platelets were further incubated with goat anti-rabbit CF^TM^488A or anti-mouse CF^TM^405M dyes for 1 h under a confocal microscope (Leica TCS SP5, Mannheim, Germany) using a 100× oil immersion objective. The intensities of immunoreaction were quantified using the NIH ImageJ software program (NIH, Bethesda, MD; http://rsbweb.nih.gov/ij/).

### Histological analysis of acute pulmonary thromboembolism in mice

Acute pulmonary microvascular thrombosis was induced per a method described by Sheu et al. [16]. A total of 48 male ICR mice were divided into four groups, each consisting of 12 mice: (1) sham-operated; (2) DMSO-treated (50 µL, 0.1%, intraperitoneal [i.p.]); (3) glabridin-treated (6 mg/kg, i.p.); and (4) glabridin-treated (12 mg/kg, i.p.). ADP (700 mg/kg) was then injected into the tail vein of each mouse, except for the sham-operated group. Ten minutes after ADP injection, the lungs were removed, fixed with 4% formalin, and embedded in paraffin. Subsequently, the lung sections were stained with hematoxylin–eosin (HE). The stained lung sections were observed, and images were obtained using Microvisioneer Manual Whole Slide Imaging (microvisioneer.com; Josef Bauer, Freising, Germany).

### Microvascular thrombus formation in mouse mesenteric vessels irradiated using sodium fluorescein

The thrombogenic method applied to the animal model in this experiment conformed to the *Guide for the Care and Use of Laboratory Animals* (8th edition, 2011), and we received an affidavit of approval for the animal use protocol from Taipei Medical University (LAC-2021-0216). Briefly, a total of 36 male ICR mice were divided into three groups, with 12 mice in each group: (1) DMSO-treated (50 µL, 0.1%, i.p.); (2) glabridin-treated (6 mg/kg, i.p.); and (3) glabridin-treated (12 mg/kg, i.p.). This was followed by the intravenous administration of sodium fluorescein (15 µg/kg), as described in another study [17]. The mice were anesthetized with intraperitoneal sodium pentobarbital (50 mg/kg). Venules (30–40 μm) were irradiated at a wavelength of < 520 nm to produce a microthrombus, and the time required for the thrombus to occlude the microvessel (occlusion time) was recorded.

### Statistical analysis

The results are expressed as the mean ± standard error of the mean and included the number of observations (*n*); *n* refers to the number of experiments, each of which was conducted using different blood donors. Significant differences between the experimental groups were analyzed using one-way analysis of variance with the Student–Newman–Keuls post hoc test to control for family-wise type I errors. A *P* value of < 0.05 indicated significance. SAS (version 9.2; SAS, Cary, NC, United States) was used for analysis.

## Results

### Concentration–response profile of glabridin in collagen-stimulated platelet aggregation

In one study [12], glabridin (Fig. 1A and 10–40 µM) most strongly inhibited human platelet aggregation by collagen (1 µg/mL) stimulation and moderately inhibited by AA (60 µM) stimulation; it did not affect platelet aggregation stimulated by thrombin (0.01 U/mL) or U46619 (1 µM). In that study [12], the authors used only three concentrations of glabridin to estimate the approximate IC_50_ value for collagen-stimulated platelet aggregation. In this study, we used six concentrations (5–30 µM) of glabridin to obtain a more accurate concentration–response profile for collagen (1 µg/mL) stimulation (Fig. 1B). No significant effects were present at concentrations < 15 µM, whereas platelet aggregation was almost completely inhibited at concentrations of 25–30 µM. The IC_50_ value was approximately 20 µM. Additionally, we conducted experiments to evaluate the potential cytotoxic effects of glabridin on human platelets. Our results showed that glabridin did not cause a significant increase in lactate dehydrogenase release within the concentration range of 20–100 µM, indicating that it is not cytotoxic to platelets (see Additional file 1: Fig. S1). Therefore, the IC_50_ and maximal (30 µM) concentrations of glabridin were subsequently used to investigate the relationship between integrin α_IIb_β_3_-mediated signals and NF-κB activation underlying collagen-stimulated platelet activation.

### Regulation of integrin α_IIb_β_3_ inside-out signals by glabridin

The collagen glycoprotein (GP) VI initiates integrin α_IIb_β_3_ inside-out signals from the intracellular SH3 binding region, which recruits the active forms of Src family kinases (SFKs) Fyn and Lyn before adhesion to collagen, causing the activation of the cytosolic tyrosine kinase Syk [18]. As shown in Fig. 1C–E, the phosphorylation of Lyn, Fyn, and Syk stimulated by collagen was inhibited by glabridin (20 and 30 µM). Integrin β_3_ can be activated by collagen and was markedly suppressed by glabridin (20 and 30 µM; Fig. 1F); corresponding statistical data are presented in the lower panels of individual immunoblotting figures.

### Relative effectiveness of glabridin compared with BAY11-7082 and Ro106-9920 on NF-κB signaling

**Fig. 2:**
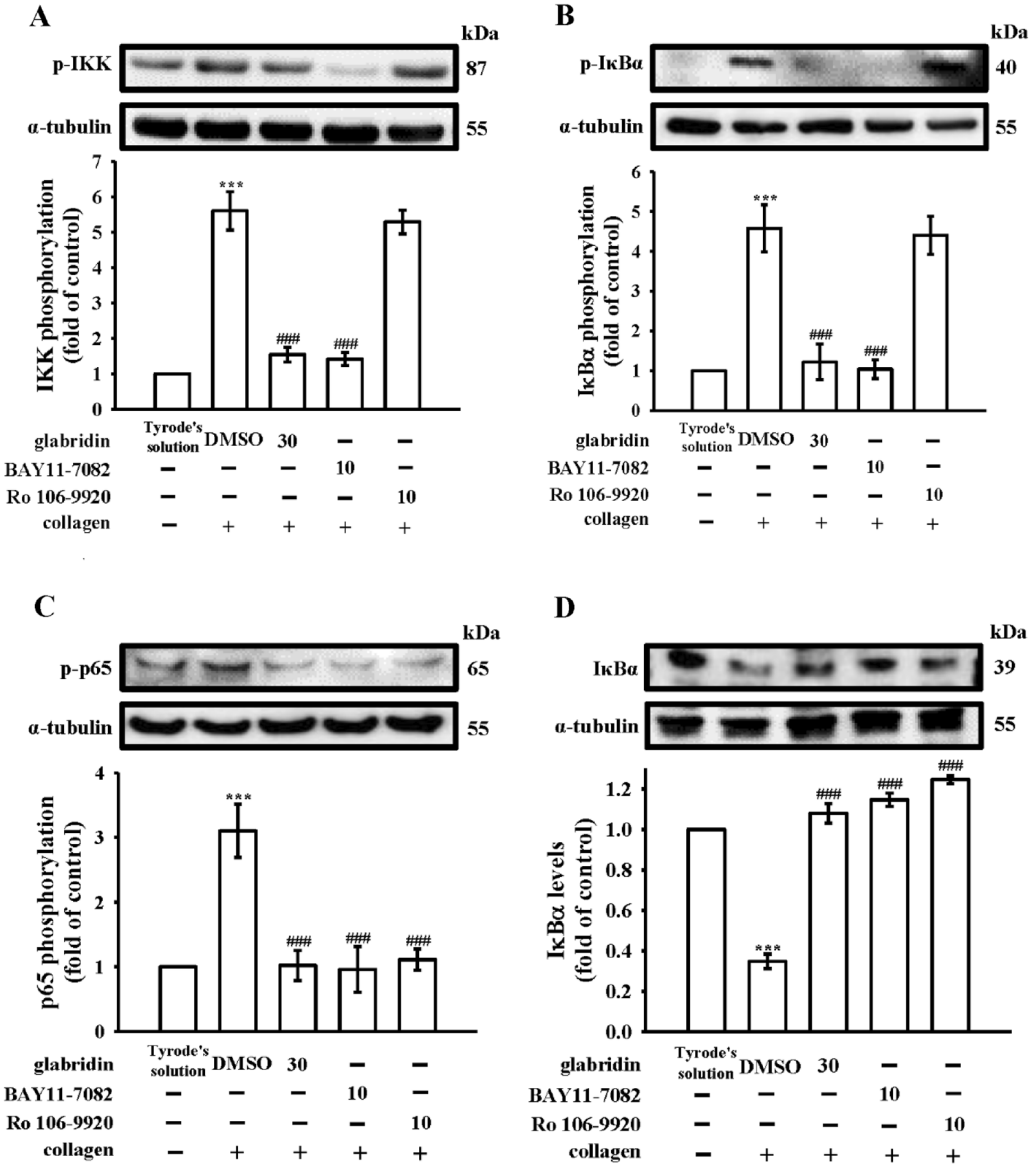
Inhibitory activity of glabridin, BAY11-7082, and Ro106-9920 on the activation of NF-κB in human platelets. Washed platelets were preincubated with 0.1% DMSO, glabridin (30 µM), BAY11-7082 (10 µM), or Ro106-9920 (10 µM), followed by the addition of collagen (1 µg/mL) to trigger NF-κB activation for the immunoblotting of **A** IKK, **B** IκBα, and **C** p65 phosphorylation as well as **D** IκBα degradation. Data are presented as mean ± standard error of the mean (*n* = 4). ***P < 0.001, compared with the resting control (Tyrode’s solution); ^###^*P* < 0.001, compared with the 0.1% DMSO-treated group

Glabridin was reported to reduce NF-κB signaling in platelets [12]. The relative effectiveness of glabridin compared with other NF-κB inhibitors (BAY11-7082 and Ro106-9920) in NF-κB activation was investigated in this study. BAY11-7082 is a representative IKK inhibitor that has prominent anticancer, neuroprotective, and anti-inflammatory effects [19]. Ro106-9920 is a selective inhibitor of the ubiquitination of activated IκBα [19]. As shown in Fig. 2, collagen markedly triggered NF-κB activation, including IKK, IκBα, and p65 phosphorylation (Fig. 2A–C) as well as IκBα degradation (Fig. 2D). Pretreatment with glabridin (30 µM) clearly reduced IKK, IκBα, and p65 phosphorylation and reversed IκBα degradation after collagen stimulation. BAY11-7082 (10 µM) exhibited similar inhibitory patterns but inhibited NF-κB activation more potently than glabridin (Fig. 2). Interestingly, Ro106-9920 (10 µM) only inhibited p65 phosphorylation and IκBα degradation (Fig. 2C, D), having no effects on IKK and IκBα phosphorylation (Fig. 2A, B). In addition, we also observed that there was no significant difference when using either α-tubulin or total protein as internal control in immunoblotting study (see Additional file 1: Fig. S2).

### Inhibitory activity of glabridin and NF-κB inhibitors using immunostaining of IKK and p65 phosphorylation and scanning confocal microscopy

**Fig. 3:**
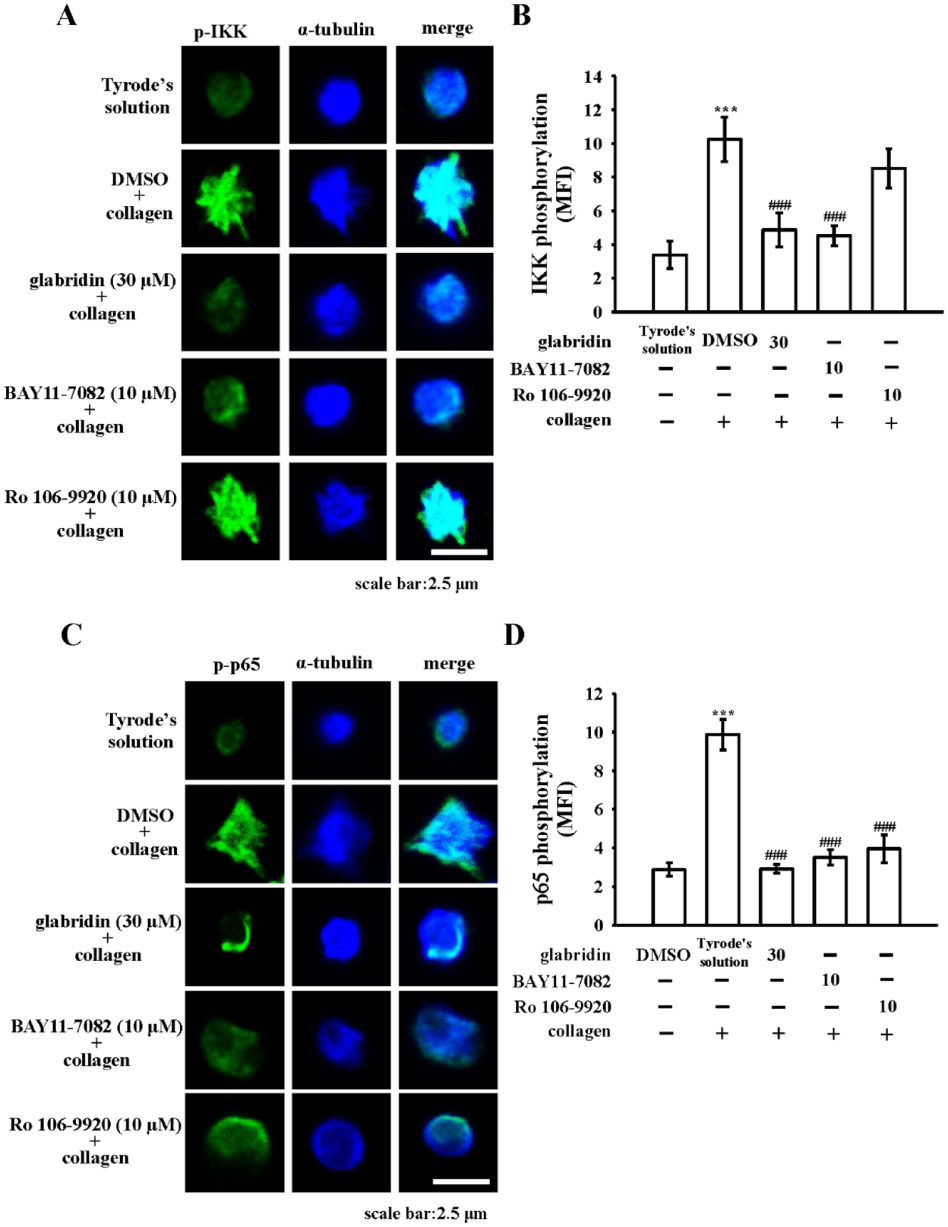
Effects of glabridin, BAY11-7082, and Ro106-9920 in regulating collagen-stimulated NF-κB activation measured using confocal laser fluorescence. Washed platelets were preincubated with 0.1% DMSO, glabridin (30 µM), BAY11-7082 (10 µM), or Ro106-9920 (10 µM), followed by the addition of collagen (1 µg/mL) to trigger platelet activation. The confocal images of **A**, **B** IKK (green fluorescence), **C**, **D** p-p65 (green fluorescence), and α-tubulin (blue fluorescence) were observed using goat anti-rabbit CF^TM^488A and goat anti-mouse CF^TM^405M dyes, respectively, as described in “Materials and methods”. The confocal images represent four similar experiments. bar: 2.5 μm. The intensity of green fluorescence representing the **A**, **B** phospho-IKK and **C**, **D** phospho-p65 were quantified in at least four different fields per view (mean fluorescence intensity [MFI]). Data are presented as mean ± standard error of the mean. ***P < 0.001, compared with the resting control (Tyrode’s solution); ^###^*P* < 0.001, compared with the 0.1% DMSO-treated group

The inhibitory activity of glabridin and NF-κB inhibitors BAY11-7082 and Ro106-9920 in IKK and p65 phosphorylation was further confirmed by using confocal laser fluorescence microscopy to compare the direct immunostaining of anti-IKK and anti-p-p65 mAbs (green fluorescence) with that of α-tubulin (blue fluorescence) in resting or collagen-activated platelets. The presence of collagen increased both p-IKK (Fig. 3A, B) and p-p65 (Fig. 3C, D) fluorescence compared with the resting group. The presence of glabridin and BAY11-7082 significantly diminished the intensity of p-IKK and p-p65. The presence of Ro106-9920 reduced the fluorescence of p-p65 but not p-IKK as compared with the control group. α-tubulin intensity did not vary among the groups. BAY11-7082 exhibited greater complete inhibiting activity than Ro106-9920 in NF-κB activation. Therefore, BAY11-7082 was used to investigate the relationship between NF-κB and integrin α_IIb_β_3_ signals in the subsequent experiments.

### Relationship between NF-κB signaling and integrin α_IIb_β_3_ inside-out signals

**Fig. 4:**
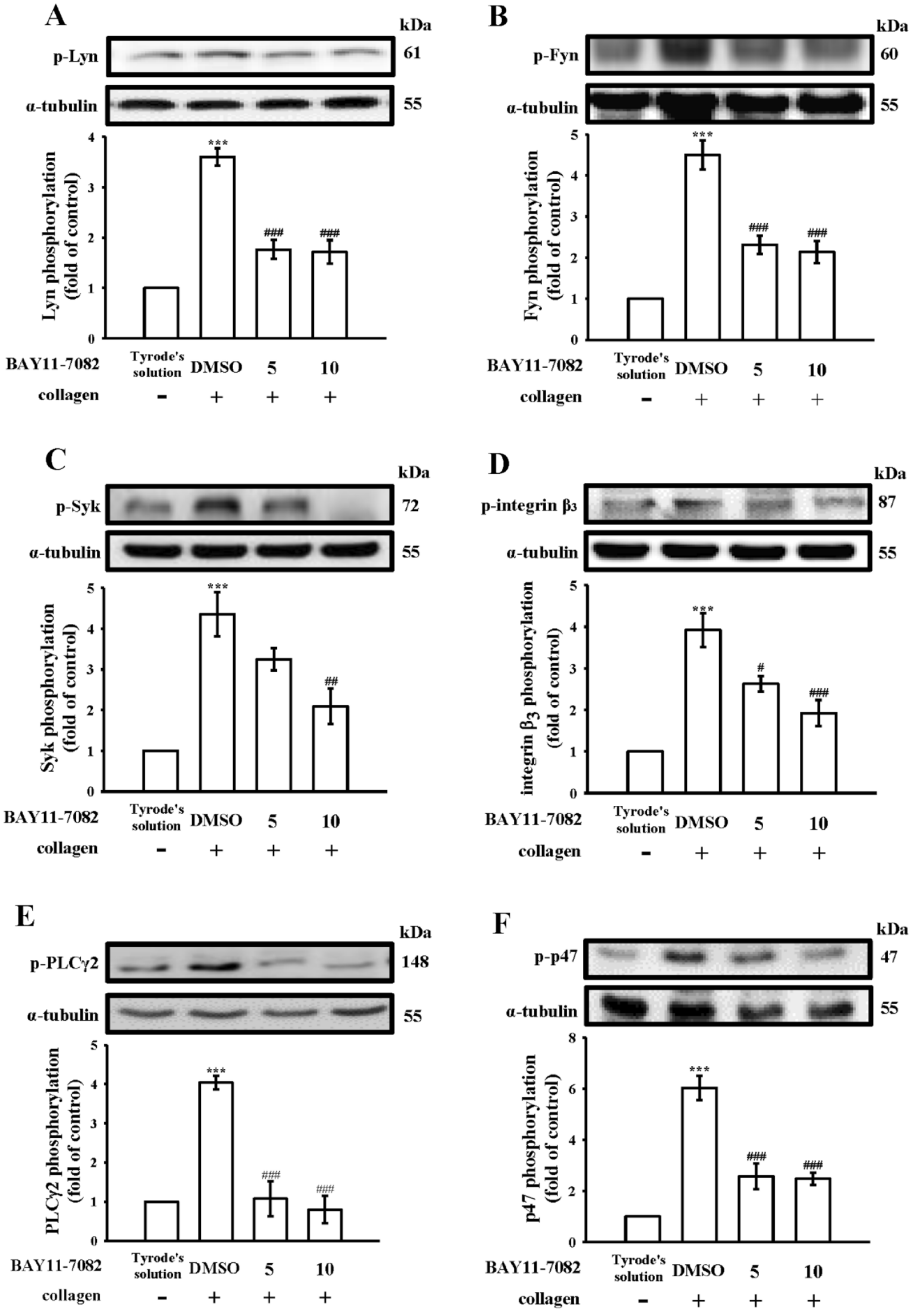
Inhibitory activity of BAY11-7082 in Lyn, Fyn, Syk, integrin β_3_, phospholipase Cγ2, and protein kinase C activation in human platelets. **A** Washed platelets were preincubated with a solvent control (0.1% DMSO) or BAY11-7082 (5 and 10 µM), followed by the addition of collagen (1 µg/mL) to stimulate **A** Lyn, **B** Fyn, **C** Syk, (D) integrin β_3_, **E** phospholipase Cγ2 (PLCγ2), and **F** protein kinase C (PKC) activation. Data are presented as mean ± standard error of the mean (*n* = 4). ***P < 0.001, compared with the resting control (Tyrode’s solution); ^#^*P* < 0.05, ^##^*P* < 0.01, and ^###^*P* < 0.001, compared with the 0.1% DMSO-treated group

**Fig. 5:**
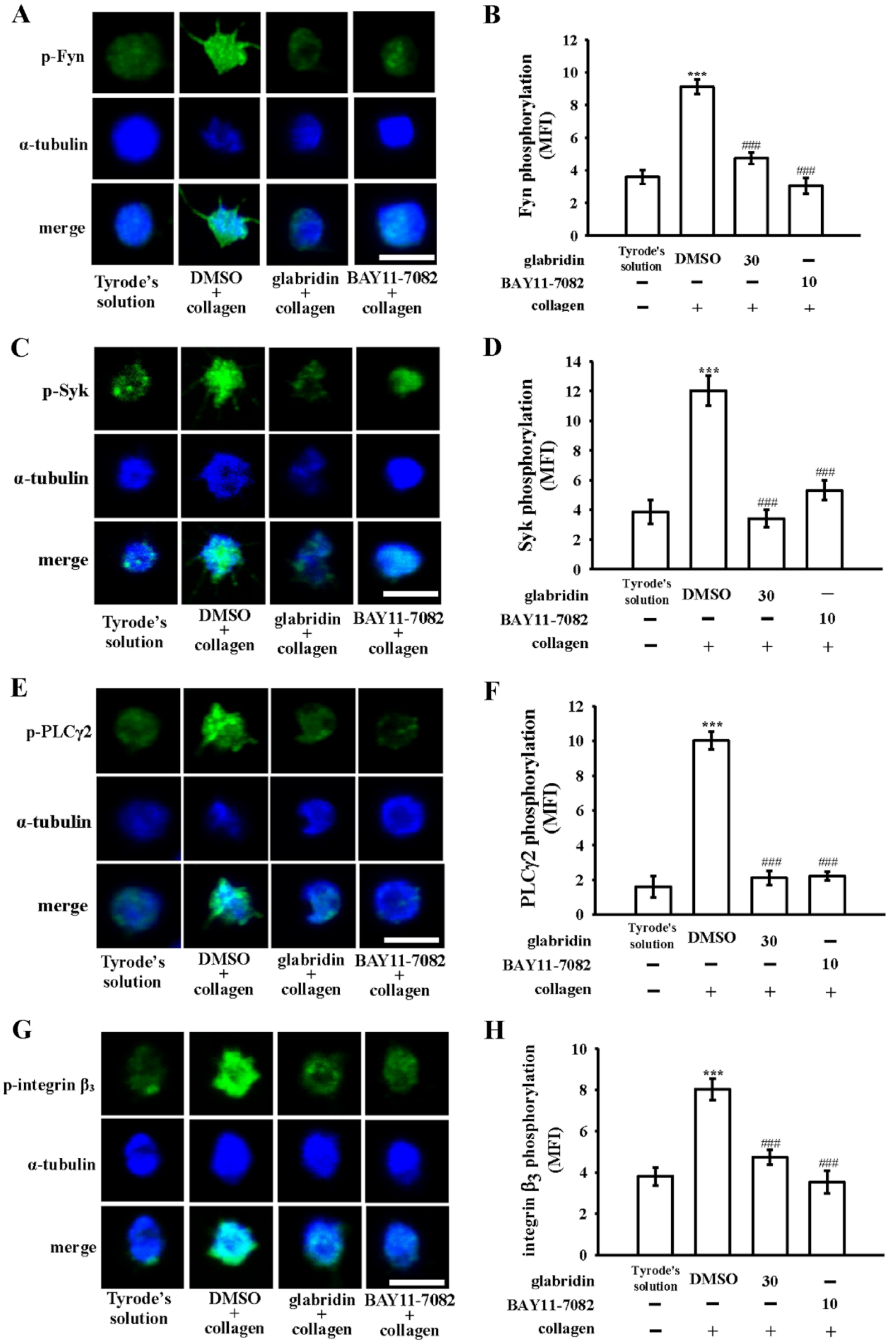
Effects of glabridin and BAY11-7082 in regulating Fyn, Syk, phospholipase Cγ2, and integrin β_3_ phosphorylation in platelet activation measured using confocal laser fluorescence. Washed platelets were preincubated with 0.1% DMSO, glabridin (30 µM), or BAY11-7082 (10 µM), followed by the addition of collagen (1 µg/mL) to trigger platelet activation. The confocal images with green fluorescence of **A**, **B** Fyn, **C**, **D** Syk, **E**, **F** phospholipase Cγ2 (PLCγ2), and **G**, **H** integrin β_3_ phosphorylation and blue fluorescence of α-tubulin were produced using goat anti-rabbit CF^TM^488A and goat anti-mouse CF^TM^405M dyes, respectively. The confocal images represent four similar experiments. bar: 2.5 μm. The intensity of green fluorescence representing the phosphorylation of **A**, **B** Fyn, **C**, **D** Syk, **E**, **F** PLCγ2 and **G**, **H** integrin β_3_ were quantified in at least four different fields per view (mean fluorescence intensity [MFI]). Data are presented as mean ± standard error of the mean. ***P < 0.001, compared with the resting control (Tyrode’s solution); ^###^*P* < 0.001, compared with the 0.1% DMSO-treated group

Platelets activated by agonists (i.e., collagen) cause a conformational change in integrin α_IIb_β_3_, facilitating ligand binding and subsequently causing activation of a series of signals such as Fyn/Lyn, Syk, and PLCγ2/PKC, and thus the onset of platelet aggregation [4]; this process is integrin α_IIb_β_3_ inside-out signaling. In this study, we further investigated cellular signaling events between integrin α_IIb_β_3_ inside-out signaling and NF-κB activation. Pretreatment with BAY11-7082 (10 µM) nearly completely inhibited collagen-stimulated Fyn, Lyn, Syk, and integrin β_3_ phosphorylation (Fig. 4A–D). PLC hydrolyzes phosphatidylinositol 4,5-bisphosphate to produce two critical secondary messengers, diacylglycerol (DAG) and inositol trisphosphate (IP_3_). DAG activates PKC, thus triggering the phosphorylation of an approximately 47-kDa protein (pleckstrin or p47) [18]. In this study, BAY11-7082 markedly reduced collagen-stimulated PLCγ2/PKC activation. Confocal examination also indicated that BAY11-7082 diminishes the fluorescence of p-Fyn, p-Syk, p-PLCγ2, and p-integrin β_3_ (Fig. 5), which suggests that NF-κB and integrin α_IIb_β_3_ inside-out signals share a mutual activation mechanism in platelet activation. Additionally, glabridin also effectively diminished all integrin α_IIb_β_3_ inside-out signals phosphorylation (Fig. 5).

### Effectiveness of antithrombotic activity by glabridin in acute pulmonary embolism and mesenteric microvessels of mice

**Fig. 6:**
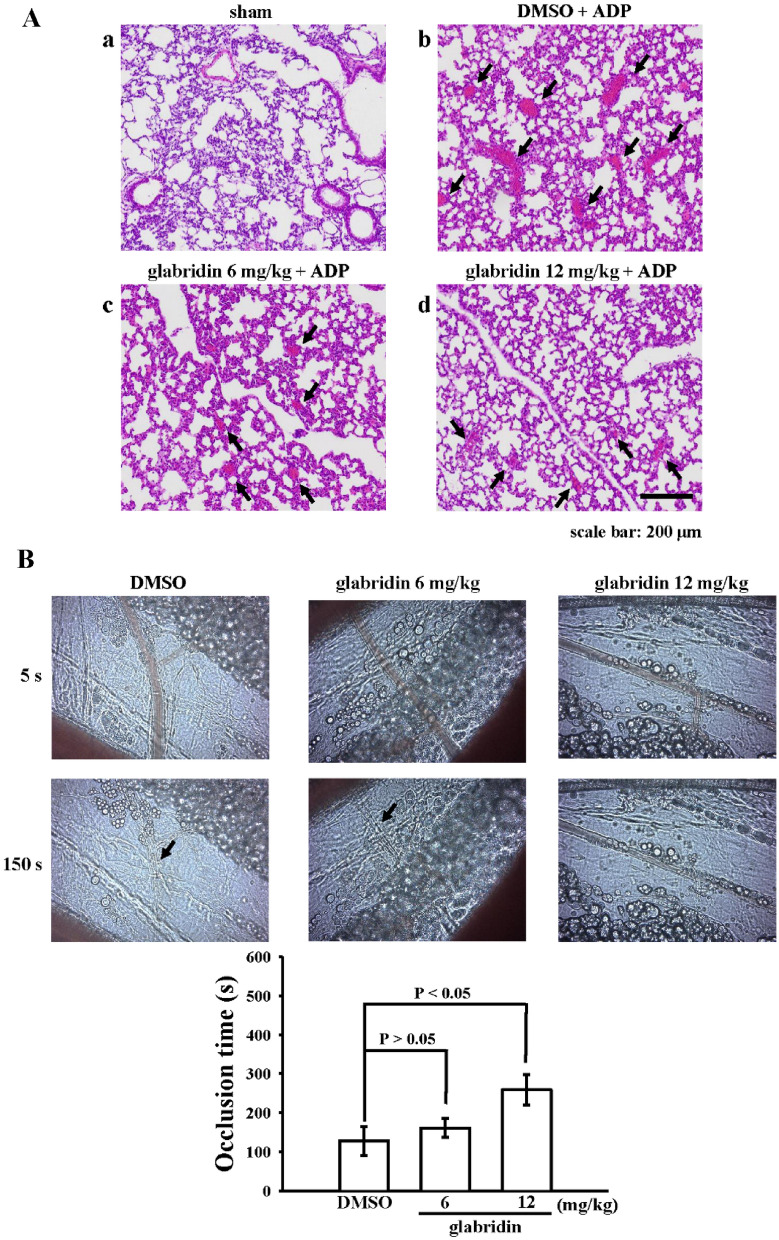
Effectiveness of glabridin on thromboembolism in lungs and platelet plug occlusion in mesenteric venules of mice. **A** To induce acute pulmonary thrombosis, 0.1% DMSO or glabridin (6 and 12 mg/kg) was intraperitoneally administered in mice, and ADP (700 mg/kg) was injected into the tail vein. Histological examination of pulmonary thrombosis (black arrows) was undertaken by staining lung tissue sections with hematoxylin–eosin as described in “Materials and methods”. **B** Mice were administered an intraperitoneal injection of solvent control (0.1% DMSO) or glabridin (6 and 12 mg/kg), and mesenteric venules were irradiated using fluorescein to induce microthrombus formation (occlusion time). Microscopic images were also recorded at 5 and 150 s after irradiation. Black arrows indicate platelet plug formation (×40 magnification). Data are presented as the mean ± standard error of the mean (*n* = 12)

The therapeutic application of glabridin was investigated as described by Chung et al. [12]. In that study, glabridin reduced mortality in acute pulmonary embolism; however, the authors did not explain the possible reasons in reduction of mortality. Therefore, we histologically examined lung slices after the intravenous injection of ADP under the same conditions and observed that a significantly higher number of lung vessels were completely or partially occluded by platelet thrombi in ADP-treated mice as compared with the sham group (Fig. 6A—a, b; black arrows). Glabridin (6 and 12 mg/kg) treatment markedly reduced the number of occluded vessels in the lungs in a dose-dependent manner as compared with 0.1% DMSO treatment (Fig. 6A—c, d). In addition, another in vivo model was employed to further verify the effectiveness of glabridin in antithrombotic activity by fluorescein-induced platelet plug formation in mesenteric microvessels. The occlusion time in the mesenteric microvessels of mice pretreated with fluorescein sodium (15 µg/kg) was approximately 130 s in 0.1% DMSO-treated mice (Fig. 6B). The occlusion time was significantly prolonged after treatment with 12 mg/kg glabridin, but not with 6 mg/kg, as compared with 0.1% DMSO treatment (DMSO, 127 ± 37 s; glabridin, 6 mg/kg, 161 ± 25 s; 12 mg/kg, 259 ± 39; *n* = 12; Fig. 6B). After irradiation, a thrombotic platelet plug was observed in mesenteric microvessels at 150 s, but not at 5 s, in the 0.1% DMSO and glabridin (6 mg/kg)-treated groups (Fig. 6B; black arrows). These results further strengthen in vivo indications of the antithrombotic applications of glabridin.

## Discussion

Licorice root is widely regarded as a valuable herbal medicine due to its exceptional pharmacological properties, which have been recognized by traditional medicine practitioners [9]. For centuries, licorice has been used in Asia and Europe as an antidote, expectorant, antioxidant, anti-inflammatory agent, as well as a flavoring and sweetening agent [20]. Glabridin, a main constituent of the hydrophobic fractions of licorice extract, is particularly noteworthy for its medicinal properties. This study supported that glabridin causes considerable antiplatelet activity in human platelets and animal experiments. The results indicated that concentrations of 20 and 30 µM were sufficient for antiplatelet activation. Although glabridin quantities obtained from natural sources would not be sufficient to achieve the necessary plasma concentration to inhibit platelet activation in humans, herbal medicines are usually administered with long-term regimens. Intake of sufficient natural ingredients or nutritional supplements is effective in preventing CVDs; thus, glabridin may serve as an innovative antithrombotic agent in clinical applications.

**Fig. 7:**
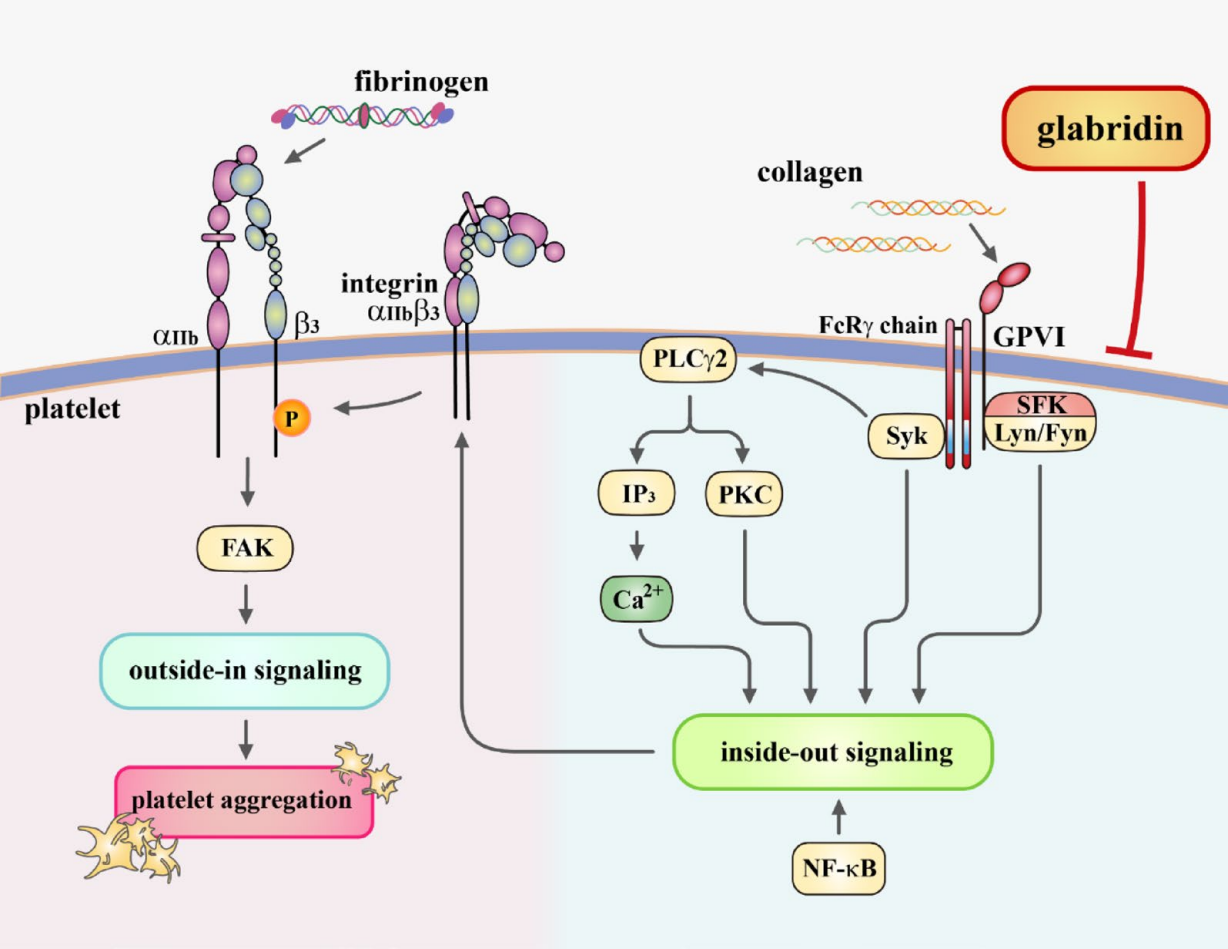
Hypothesis regarding inhibitory mechanisms of glabridin in platelet activation. Glabridin affects integrin α_IIb_β_3_ inside-out signaling and NF-κB activation, causing inhibition of platelet aggregation

Platelet activation can be induced by various physiological stimuli such as collagen and thrombin. These stimuli can act through specific receptors or by modifying signal transduction pathways associated with other receptors. Upon activation, platelets undergo a conformational change in integrin α_IIb_β_3_, which promotes the binding of ligands such as fibrinogen, von Willebrand factor, and fibronectin, leading to platelet aggregation. This process is known as integrin α_IIb_β_3_ inside-out signaling (Fig. 7) [4]. After fibrinogen binds to integrin α_IIb_β_3_, integrin α_IIb_β_3_-mediated signaling is initiated, which triggers the tyrosine phosphorylation of several proteins, including focal adhesion kinase (FAK) and integrin β_3_. This process relies on outside-in signaling and cytoskeleton reorganization (Fig. 7) [4, 21]. Triflavin, a disintegrin containing Arg-Gly-Asp, acts as a specific antagonist to integrin α_IIb_β_3_ by inhibiting fibrinogen-integrin α_IIb_β_3_ interaction [22]. Triflavin can inhibit platelet aggregation stimulated by various agonists, including collagen, thrombin, ADP, and U46619 [23]. These results suggest that Triflavin establishes a common inhibitory pathway instead of acting on individual agonist receptors [12]. Glabridin effectively inhibited collagen-stimulated activity; nevertheless, it had no effects on thrombin- or U46619-stimulated activity, indicating that glabridin may not affect the binding of fibrinogen-integrin α_IIb_β_3_ (outside-in signals). Generally, signaling processes occurring during platelet activation can be classified into several stages: the interaction of agonists with their individual receptors, which mediates platelet early activation, followed by common signaling events (i.e., PLCγ2, MAPK, and NF-κB), and integrin α_IIb_β_3_ activation including inside-out (i.e., Lyn, Fyn, and Syk activation) and outside-in signaling (platelet spreading and fibrin clot retraction) [24]. Platelet activation is a dynamic process involving feedback loops and crosstalk between pathways.

Following vascular injury, platelet adhesion and aggregation are triggered by the exposure of subendothelial collagen at the injury site, leading to vascular thrombosis. Collagen is present in the subendothelial space and within the tunica media of blood vessels, making it the most crucial protein that interacts with platelets and induces activation responses. Among collagen receptors, GP VI is believed to play a key role and is a necessary factor for platelet aggregate formation on the collagen surface under blood flow. GP VI is a membrane protein in the immunoglobulin superfamily that forms a complex with the Fc receptor γ-chain containing immunoreceptor tyrosine-based activation motifs and is phosphorylated by SFKs, such as Fyn and Lyn [25]. Subsequently, various protein phosphorylation pathways, including Syk, regulate integrin α_IIb_β_3_ activation through inside-out mechanisms (Fig. 7). Additionally, the vital role of the cytoplasmic tail of integrin β_3_ phosphorylation stimulated by immobilized fibrinogen in platelets was validated in vivo, and its mutation led to bleeding disorder and strongly affected clot retraction in vitro [26]. In addition to immobilized fibrinogen, we found that collagen could activate integrin β_3_ phosphorylation, which clearly demonstrates that collagen stimulates integrin β_3_ inside-out signals and subsequently influences integrin β_3_ phosphorylation. In this study, glabridin markedly diminished Fyn, Lyn, and Syk and integrin β_3_ phosphorylation stimulated by collagen, indicating that glabridin inhibits platelet aggregation through integrin β_3_ inside-out signals.

NF-κB’s role in nucleated cells is well-established, as it has been extensively studied. When triggered by stimuli such as cytokines, ultraviolet radiation, or free radicals, NF-κB is activated and can cause inflammation, as well as impair cell survival, differentiation, and proliferation [27]. In human atherosclerotic plaques, activated NF-κB is responsible for the development of unstable coronary plaques [28]. Despite the lack of nuclei in platelets, they contain NF-κB, which is involved in platelet activation, independent of genomic functions. Using immunoblotting assays and a confocal microscope, it has been demonstrated that glabridin inhibits NF-κB activation, which includes IKK, IκBα, and p65 phosphorylation, as well as IκBα degradation. Thus, NF-κB signaling plays a significant role in glabridin’s antiplatelet activity. Furthermore, it has been reported that NF-κB inhibitors decrease platelet activation [29]. In this study, BAY11-7082 more strongly inhibited integrin β_3_ inside-out signals than Ro106-9920, and these signals are critical in glabridin-mediated antiplatelet activity.

Animal models are crucial in understanding the therapeutic significance of test drugs in various diseases. To further evaluate the in vivo efficacy of glabridin against vascular thrombosis, platelet plug formation in mesenteric microvessles [17] was performed in this study. Mesenteric venules were continuously irradiated with fluorescein throughout the experiment, causing considerable injury of endothelial cells and platelet plug formation. Treatment with galbridin significantly extended the occlusion time. These data are consistent with the fact that platelet aggregation is a critical factor for vascular thrombosis. In histological analysis of acute pulmonary thromboembolic mice, we observed that a substantially high number of lung vessels were completely or partially occluded by platelet thrombi after injection of ADP; glabridin effectively reduced the occluded lung vessels. Therefore, glabridin is a potential natural treatment for thromboembolic disorders.

## Conclusion

This study identified glabridin as a potential antithrombotic agent that blocks integrin α_IIb_β_3_ inside-out signaling in human platelets, as depicted in Fig. 7. The study provided further insights into the role of glabridin in preventing CVDs. However, we cannot rule out the possibility that other unidentified mechanisms may have been involved in glabridin-mediated antiplatelet activity.

## Supplementary Information

**Additional file 1: Figure S1.** Effects of glabridin on cytotoxicity in human platelets. Washed human platelets were pretreated with either the solvent controlor glabridinfor 20 min, and a 10 μL of the supernatant was dropped on a Fuji Dri-Chem slide LDH-PIII. Data are presented as the mean ± standard error of the mean. **Figure S2.** Inhibitory profiles of glabridin for IKK, p65, Lyn and integrin β_3_ activation stimulated by collagen in human platelets. Washed platelets were preincubated with a solvent controlor glabridin, followed by the addition of collagento stimulateIKK,p65,Lyn, andintegrin β_3_ phosphorylation.

## Data Availability

Data analyzed or generated during this study are included in this manuscript.

## Abbreviations

ADP: Adenosine diphosphate
BAY11-7082: 3-[(4-Methylphenyl)sulfonyl]-(2E)-propenenitrile
BSA: Bovine serum albumin
CVDs: Cardiovascular diseases
DAG: Diacylglycerol
DMSO: Dimethyl sulfoxide
EDTA: Ethylenediaminetetraacetate
GSK3β: Glycogen synthase kinase-3β
GP: Glycoprotein
IP3: Inositol trisphosphate
LDL: Low-density lipoprotein
MAPK: Mitogen-activated protein kinase
PFA: Paraformaldehyde
PMSF: Phenylmethylsulfonyl fluoride
PBS: Phosphate-buffered saline
PLCγ2: Phospholipase Cγ2
PRP: Platelet-rich plasma
PKC: Protein kinase C
Ro106-9920: 6-(Phenylsulfinyl)-tetrazolo[1,5-b]pyridazine
NaF: Sodium fluoride
SFKs: Src family kinases
U46619: 9,11-Dideoxy-9α,11α-methanoepoxy-prosta-5Z,13E-dien-1-oic acid
