# Supplementary material for: Mechanisms of glabridin inhibition of integrin αIIbβ3 inside-out signals and NF-κB activation in human platelets
Source: Chin Med. 2023 Jun 10;18:71. doi: 10.1186/s13020-023-00779-9 (PMC10257322; doi:10.1186/s13020-023-00779-9)
Supplement: Supplementary file 1 — Additional file 1: Figure S1. Effects of glabridin on cytotoxicity in human platelets. Washed human platelets were pretreated with either the solvent controlor glabridinfor 20 min, and a 10 μL of the supernatant was dropped on a Fuji Dri-Chem slide LDH-PIII. Data are presented as the mean ± standard error of the mean. Figure S2. Inhibitory profiles of glabridin for IKK, p65, Lyn and integrin β3 activation stimulated by collagen in human platelets. Washed platelets were preincubated with a solvent controlor glabridin, followed by the addition of collagento stimulateIKK,p65,Lyn, andintegrin β3 phosphorylation. [file 13020_2023_779_MOESM1_ESM.pdf]

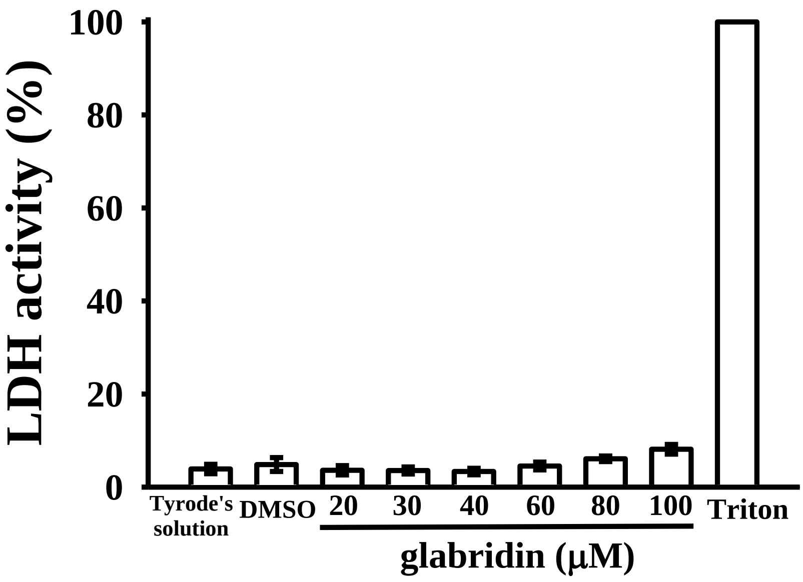

Figure S1. Effects of glabridin on cytotoxicity in human platelets. Washed human platelets were pretreated with either the solvent control (0.1% DMSO) or glabridin (20-100 μM) for 20 mins, and a 10 μL of the supernatant was dropped on a Fuji Dri-Chem slide LDH-PIII. Data are presented as the mean ± standard error of the mean (n= 3).

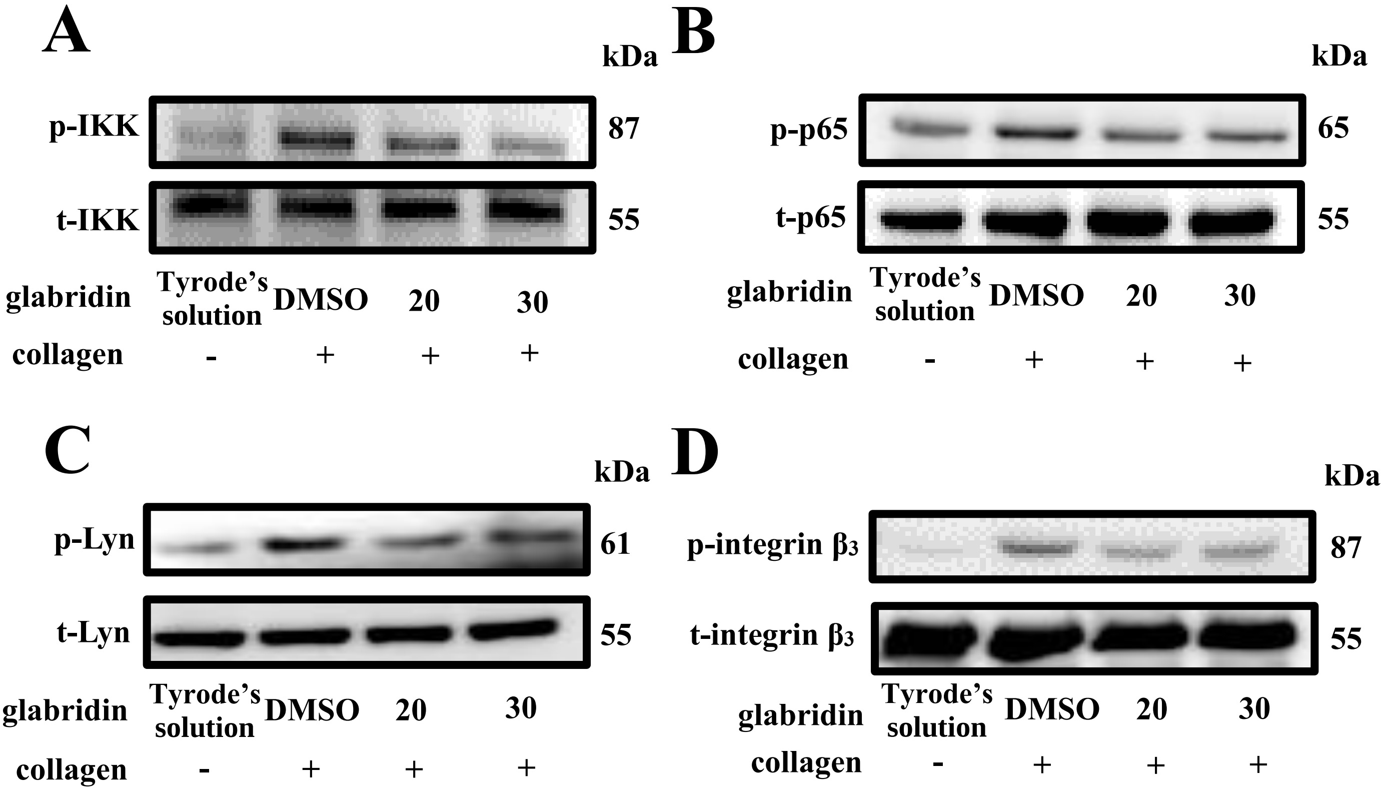

Figure S2. Inhibitory profiles of glabridin for IKK, p65, Lyn and integrin β_3 activation stimulated by collagen in human platelets. Washed platelets were preincubated with a solvent control (0.1% DMSO) or glabridin (20 and 30 μM), followed by the addition of collagen (1 μg/mL) to stimulate (A) IKK, (B) p65, (C) Lyn, and (D) integrin β_3 phosphorylation (n= 3).
